# One-Pot, Optimized Microwave-Assisted Synthesis of Difunctionalized and B–N Co-Doped Carbon Dots: Structural Characterization

**DOI:** 10.3390/nano13202753

**Published:** 2023-10-12

**Authors:** Hector Daniel Ibarra-Prieto, Alejandra Garcia-Garcia, Faustino Aguilera-Granja, Diana Carolina Navarro-Ibarra, Ignacio Rivero-Espejel

**Affiliations:** 1 Centro de Investigación en Materiales Avanzados, S.C. (CIMAV), Subsede Monterrey, Av. Alianza Norte 202, Parque PIIT, Apodaca 66628, Nuevo León, Mexico; 2 Grupo de Síntesis y Modificación de Nanoestructuras y Materiales Bidimensionales-CIMAV, Subsede Monterrey, Monterrey 66628, Nuevo León, Mexico; 3 Instituto de Física “Manuel Sandoval Vallarta”, Universidad Autónoma de San Luis Potosí, Álvaro Obregón 64, San Luis Potosí 78000, San Luis Potosí, Mexico; 4 Tecnológico Nacional de Mexico, Instituto Tecnológico del Valle de Etla, Abasolo S/N, Barrio del Agua Buena, Santiago Suchilquitongo 68230, Oaxaca, Mexico; 5 Centro de Graduados e Investigación, Instituto Tecnológico de Tijuana, Tijuana 22000, Baja California, Mexico

**Keywords:** carbon dots, co-doped carbon dots, difunctionalized carbon dots, one pot, N–B co-doped carbon dots

## Abstract

In this work, we employed a novel microwave-assisted synthesis method to produce nitrogen and boron co-doped carbon dots (B–N co-doped CDs). To achieve optimal synthesis, we conducted a comprehensive parameter modulation approach, combining various synthesis temperatures, times, and precursor concentrations, while keeping the power constant at 150 W and pH 5. Using maximum fluorescence emission as our response variable, the best conditions were identified as 120 °C, 3 min, and a precursor concentration of 1 mg/mL. Characterization using field emission scanning electron microscopy revealed these CDs to have a spherical morphology with an average size of 10.9 ± 3.38 nm. Further high-resolution transmission electron microscopy showed an interplanar distance of 0.23 nm, which is in line with prior findings of CDs that present a 0.21 nm distance corresponding to the (100) plane of graphite. Optical properties were ascertained through UV–vis absorption, identifying distinct π–π* and n–π* transitions. Fluorescence spectroscopy highlighted an emission peak at 375 nm when excited at 295 nm, achieving a quantum yield of 56.7%. Fourier-transform infrared spectroscopy and Raman spectroscopy analyses confirmed the boronic acid and amine groups’ presence, underscoring the graphitic nature of the core and the co-doping of boron and nitrogen. These empirical observations were compared with theoretical investigations through simulated Raman spectra, proposing a potential structure for the CDs. X-ray photoelectron spectroscopy further endorsed the co-doping of nitrogen and boron, along with the detection of the specified functional groups. All these characteristics could lend this nanomaterial to different types of applications such as fluorescent probes for a broad range of analytes and for fluorescent cell imaging.

## 1. Introduction

Carbon dots (CDs) have recently emerged as a promising class of zero-dimensional nanomaterials. These materials are composed of sp^2^ and sp^3^ carbon atoms arranged in graphitic-like architectures or amorphous polymeric chains confined into spheric particles [1,2,3,4]. CDs have garnered significant attention due to their unique optical and electronic properties, biocompatibility, and potential for various applications in fields such as biomedicine [5], sensing [6], and energy conversion [7].

The optical properties of CDs, such as their high photoluminescence quantum yield, tunable fluorescence emission, and excitation-dependent behavior, have led to their use as fluorescent sensors for different analytes [8,9,10,11,12,13,14,15] and in vitro and in vivo fluorescent cellular imaging probes [9,16,17,18]. Furthermore, CDs have shown potential in controlled drug delivery [19,20], anticancer therapy [18,21,22,23], solar cells [24,25,26], and, recently, as endoplasmic reticulum in situ antioxidants [27]. Several methods have been developed for the synthesis of CDs, including arc discharge [28], electrophoresis [29], hydrothermal synthesis [2,30,31,32], electrochemical methods [33,34], ultrasonic-assisted synthesis [35], and microwave-assisted synthesis [36,37,38].

For example, Mansour et al. reported the microwave-assisted synthesis of CDs using a high-concentrated L-cysteine solution in a multimodal microwave reactor for 60 s at 800 W [37]. While their method was successful in producing CDs, a lack of control over the parameters of synthesis led to different size distributions and forms of the nanoparticles and the formation of large precipitates even after dialysis was performed.

Researchers have turned to innovative approaches such as monomodal microwave reactors to overcome the limitations of traditional synthesis methods. This method offers several advantages, such as rapid heating and cooling, improved reaction efficiency, and precise control over reaction conditions. Microwave reactors have been demonstrated to be highly effective in synthesizing high-quality CDs with precise control over their size, morphology, and physicochemical properties [39,40].

A doping method with heteroatoms is used to enhance the physicochemical properties of the CDs. This method involves the introduction of non-carbon atoms, such as nitrogen, sulfur, or phosphorus, into the carbon lattice of CDs [41]. This process can modify the electronic structure and bandgap of the CDs, resulting in enhanced absorption and emission properties, increased conductivity, and improved stability [14]. Specifically, the addition of nitrogen and boron as dopants brings extra benefits. Firstly, nitrogen as a dopant adds free electrons to the carbon network, enhancing the conductivity properties due to its n-type dopant nature [42]. Otherwise, boron, due to its lower valence than carbon, acts like a p-type dopant in the network, which can lead to reactive sites for catalytic chemical sensing [43].

The addition of both nitrogen and boron to CDs has been studied recently. Typically, hydrothermal methods have been used to develop B and N co-doped CDs with different types of precursors, such as 3-aminophenylboronic acid [44] and boric acid/tieguanyin tea residues [45], with treatments of 8 h at 160 °C and 6 h at 220 °C, respectively. Microwave-assisted techniques have been used to achieve the preparation of B and N co-doped CDs with softer conditions. These previous works were developed using citric acid, urea, or ethylenediamine as a nitrogen source and boric acid as a boron source. All of the studies that applied household multimodal microwave ovens with treatment times from 4 to 60 min, at 160 °C to 200 °C, and using 720 to 900 W of power, led to the development of B and N co-doped CDs with QY from 7 to 66.5% [46,47,48,49].

In this work, we present a one-pot, highly controlled, fast, and reproducible microwave-assisted synthesis of difunctionalized and B–N co-doped CDs utilizing a sole precursor as the carbon and functional group source and water as a solvent in a monomodal microwave reactor. In addition, we discuss the parameter modulation of the method, varying the time, temperature, power, and concentration of synthesis to obtain the optimal parameters to accomplish reproducibility. Finally, the structural and optical properties of CDs were studied.

## 2. Experimental Section

### 2.1. Microwave-Assisted Synthesis of the B–N Co-Doped CDs

The difunctionalized, B–N co-doped CDs were prepared by the adaptation and modification of the hydrothermal method described by Shen and co-workers [32]. A variable amount of 3-aminophenylboronic acid monohydrate (3APBA) was mixed in ultrapure water (18.2 MΩ·cm^−1^) to obtain concentrations of 1, 3, 5, and 10 mg/mL, and the pH was modulated to 5. The solution was sonicated until complete dispersion and then placed on the microwave reactor (CEM Discover 2.0).

### 2.2. Determination of the Quantum Yield

The quantum yields (QYs) of the B–N co-doped CDs were obtained using the slope method [50]. Four different solutions of 4-methylumberipherone QY = 22% were prepared in ethanol and their absorbances were less than 0.1 at 300 nm. The QY was calculated using the following equation:

(1) $$\phi_{n}=\phi_{s}\frac{{Grad}_{n}}{{Grad}_{s}}\frac{{{(\eta }_{n})}^{2}}{{{(\eta }_{s})}^{2}}$$

where *ϕ* is the quantum yield, Grad is the slope of the correlation between the absorbance and the integrated fluorescence emission, and *η* is the refractive index of the solvent. The subscript “*n*” refers to the sample and “*s*” to the standard.

### 2.3. Modulation of Synthesis Parameters

Different iterations of the experiment were conducted to determine the optimum synthesis parameters for the B–N co-doped CDs. Firstly, with previous standard parameters (160 °C, 7 min, 150 W) four amounts of concentration were used to determine maximum PL intensity. Afterward, Table 1 shows a correlation between different values of time and temperature. The power, concentration, and pH remained constant.

An experiment design was developed with the PL of the nanomaterial as the response variable, and the decision was made after the maximum PL intensity of all the samples to optimize the parameters for the reaction.

### 2.4. Purification Process of the B–N Co-Doped CDs

After cooling to room temperature, the dispersion was passed through a cotton-filled column to remove non-reacted precursor and then centrifuged at 10,000 rpm to remove large precipitates. Further, the dispersion was dialyzed in 500–1000 Da bag tubes for 2 cycles of 30 min each at 6000 rpm in a centrifuge. Finally, the B–N co-doped CDs were preserved at 4 °C.

### 2.5. Characterization

After the obtention of the optimal parameters, all the characterizations were conducted with the sample obtained using the following conditions: 3 min, 120 °C, 150 W, and 1 mg/mL. Ultraviolet–visible spectroscopy was performed in a Cary 50 (Varian, Palo Alto, CA, USA). The photoluminescence (PL) studies were conducted on a Cary Eclipse Spectrophotometer (Varian, Palo Alto, CA, USA). Fourier-transform infrared (FTIR) was measured by letting a droplet of the dispersion dry on a PE-983 FTIR Spectrophotometer (Perkin Elmer Inc. Waltham, MA, USA). X-ray photoelectron spectroscopy (XPS) spectra were collected from an ESCALAB 250 Xi XPS (Thermo Fischer Scientific inc. Waltham, MA, USA). High-resolution transmission electron microscopy (HRTEM) measurements were performed using a JEM-2200FS (JEOL Ltd. Akishima Tokyo, Japan) and transmission electron microscopy (TEM) was conducted using a Hitachi 7700 (Hitachi Ltd. Hitachi, Ibaraki, Japan); both procedures were conducted by depositing a drop on a copper grid and evaporating the solvent. Size distribution was obtained with different TEM images with a sample of over 130 particles. Raman measurements were carried out using a LabRAM HR Evolution Raman spectrometer with a 325 nm laser (HORIBA Ltd. Minami-ku, Tokyo, Japan).

### 2.6. Theoretical Calculations

Theoretical calculations were performed by employing density functional theory (DFT) analyses focused on the B–N co-doped CDs, leveraging the computational capabilities of the GAUSSIAN09 software suite [51]. The study was primarily directed towards extracting Raman spectra and characterizing electronic and structural properties.

Different structural arrays, constructed based on previous spectroscopical results with 10 nitrogen atoms, 7–8 boron atoms, 4 oxygen atoms (considered the boronic acid groups), 44 carbon atoms, and 26 hydrogen atoms, for a total of 91–92 atoms, were subjected to optimization processes allowing the derivation of equilibrium configurations associated with electronic and structural properties. Two of the most representative arrays were B–N-LALN2DZ and Pyrrole-LALN2DZ, which were tailored to fit the chemical composition of the structures with vacancies, B–N bonds, pyrrolic nitrogen, and functional groups in the surroundings of the particle. The insights gleaned from these optimized motifs were subsequently compared with experimental data of the nanomaterial. The rest of the structural motifs will be analyzed in depth in a further vibrational study of B–N co-doped CDs.

The different structural motifs are minimized at the DFT level of the Kohn–Sham equations. The LANL2DZ basis set was utilized to solve these equations, originally proposed by Hay and Wadt [52], in conjunction with the Perdew–Burke–Ernzerhof (PBE) formulation for the exchange–correlation functional [53]. This combination delivered a desirable balance between computational efficiency and precision, particularly during the execution of fully unrestricted spin-polarized structural optimizations. The selection of the PBE functional was substantiated by consulting the relevant references in the current literature, which demonstrate its effectiveness in analyzing interactions among graphene sheets, boron, and nitrogen atoms. Zhang et al. [54] further validated the method’s competence in accurately rendering these atoms’ electronic and magnetic properties.

Upon acquiring the Raman spectra, a detailed comparison was made with the experimental results. The resulting stick spectra were broadened using a Gaussian line shape function, implementing a full width at half the maximum height of 10 cm^−1^. This process enhanced the clarity of the spectra visualization.

## 3. Results and Discussion

### 3.1. Field Emission Scanning Electron Microscopy and High-Resolution Transmission Electron Microscopy

**Figure 1:**
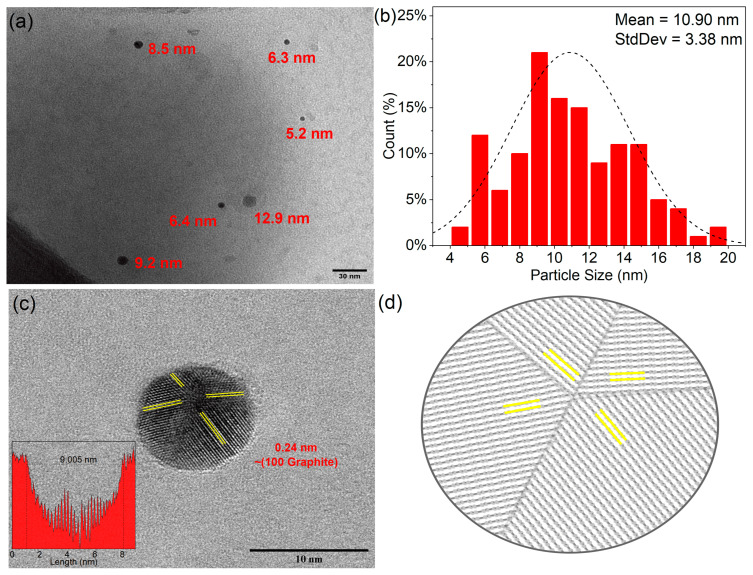
(**a**) TEM micrography of the B–N co-doped CDs synthetized using the following conditions: 120 °C, 3 min, 150 W, and 1 mg/mL; (**b**) size distribution of the B–N co-doped CDs; (**c**) HRTEM image showing the 0.24 nm planar distance in three different orientations of B–N co-doped CDs synthetized using the following conditions: 120 °C, 3 min, 150 W, and 1 mg/mL; (**c**) inset: particle profile plot showing the distance between planes and the spherical-like nature of the particle; and (**d**) proposed scheme of the arrangement of the layers within the B–N co-doped CDs.

The TEM micrograph depicted in Figure 1a illustrates a homogeneous distribution of quasi-spherical particles with a mean diameter of 10.9 ± 3.38 nm (Figure 1b). This quasi-spherical form is attributed to the tendency of nanostructures to minimize their surface and interfacial energies, resulting in agglomerates with round-like shapes [55]. Nevertheless, graphitic nanostructures are not perfectly round-shaped, and this could be attributed to the functional groups and the heteroatomic co-doping within the sample, which induces modification in the particle’s crystalline structure and, overall, in its morphology as explained in the TEM section [56].

Moving forward to the HRTEM image portrayed in Figure 1c, it represents a single N–B co-doped CD. The diameter of the displayed particle measures 9.2 nm. Moreover, a meticulous examination reveals four distinct lattice orientations with an inner-lattice spacing of 0.24 nm. 

Conventional studies [57] often report a 0.21 nm inter-lattice spacing corresponding to the graphite orientation (100). Nonetheless, the slight disparity in our case can be attributed to the incorporation of nitrogen and boron atoms within the lattice structure, thereby provoking electrostatic repulsion, which is consistent with previous works of doped CDs. Further inspection of the surroundings exposes amorphous carbon, which is synonymous with the functional groups bonded to the particle. All these characteristics are consistent with the evidence reported by Endo and coworkers [56], where they explain the co-existence of three different types of stacking in graphitic-like carbon nanostructures. Graphitic stacking, glide plane stacking, and rotational turbostratic stacking can be found in this kind of nanomaterial, leading to a single perfect graphitic crystalline structure. In the case of CDs, the presence of boron and nitrogen as co-dopants leads to rotational turbostratic stacking, functional groups such as boronic acids and amine promote glided plane stacking. This leads to a single graphitic crystalline structure (0.24 nm (100) plane).

A detailed size profile was performed, which is illustrated in Figure 1c inset. Here, measurements of the distance between the lattices were undertaken. The observed consistent inter-lattice spacing of 0.24 nm reaffirms the data from the HRTEM analysis. The profile’s depth indicates the particle’s spherical form, which serves as a differential factor between bidimensional graphene quantum dots and carbon dots. A spherical form, as demonstrated in our study, is suggestive of carbon dots. This aspect helps distinguish CDs from their graphene counterparts, which typically assume a planar or slightly curved structure. Figure 1d presents the proposed schematic representation of the particle’s growth process.

### 3.2. UV–Vis Spectroscopy and Fluorescence

**Figure 2:**
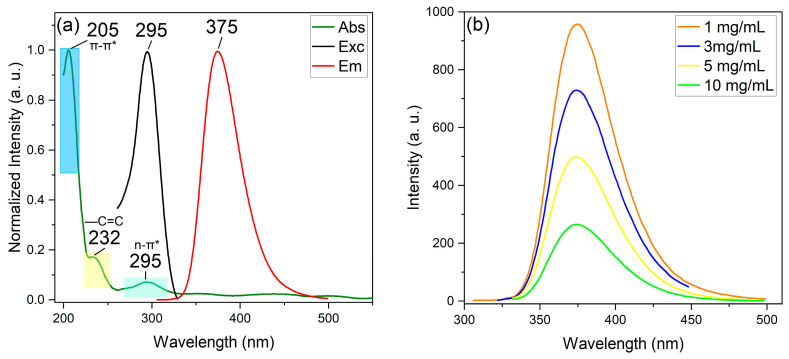
(**a**) UV–vis/fluorescence spectra of the B–N co-doped CDs synthetized using the following conditions: 120 °C, 3 min, 150 W, and 1 mg/mL. The absorbance results show the three characteristic absorbance bands (green light), the excitation band corresponding to the n–π* band of absorbance (black line), and the emission band. (**b**) Fluorescence emission at different synthesis concentrations of 3APBA.

The N–B co-doped CDs were subjected to spectroscopic analysis due to their remarkable optical properties. Initially, a UV–vis spectroscopy scan was conducted, revealing three bands at 205 nm, 232 nm, and 295 nm. These bands were attributed to π–π*, π–π*, and n–π* transitions, respectively (Figure 2a, blue, yellow, and green boxes, respectively) [58].

The interaction between the aromatic groups within the CD network explains the first band. At the same time, the second is attributed to extended conjugation (–C=C) within the carbon network of the CDs. The third band is due to the flow of electrons from the amine groups that functionalize the CD surfaces towards the carbon structure with extended conjugation within the network. The n–π* transition corresponded with the excitation band obtained through fluorescence spectroscopy. When excited at this wavelength (295 nm), the material emits a band at 375 nm (Figure 2a, black and red lines) with a QY of 57.6% [59].

### 3.3. Parameter Modulation

**Figure 3:**
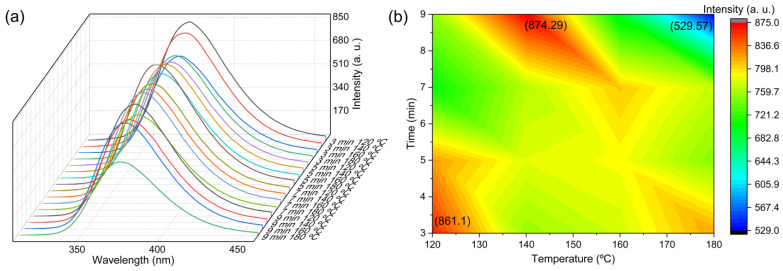
(**a**) Fluorescence emission at different times of 3 min, 5 min, 7 min, and 9 min and temperatures of 120 °C, 140 °C, 160 °C, and 180 °C, and (**b**) heat map of the maximum fluorescence emission at different times and temperatures.

During the synthesis of boron and nitrogen co-doped carbon dots (N–B co-doped CDs), it is essential to optimize various parameters to obtain the desired optical properties. One such parameter is the amount of sole precursor used, which was varied in the experiments conducted to optimize CD synthesis. The fluorescence spectra of the CDs were obtained using different concentrations of 3APBA, namely, 1, 3, 5, and 10 mg/mL. The results showed that the fluorescence intensity decreased as the initial solution concentration increased (Figure 3b) and can be attributed to the possibility of obtaining larger particle sizes and aggregation, combined with an amount of unreacted precursor, resulting in a decrease in the optical property.

On the other hand, lower concentrations can provide better control over the size of the N–B co-doped CDs, promote particle dispersion in the solvent, and avoid waste of unreacted precursors, as evidenced by increased fluorescence intensity. Thus, the optimum precursor concentration to obtain the desired optical properties of CDs is 1 mg/mL.

In addition to the precursor concentration, temperature and reaction time are other parameters that need to be optimized in the synthesis of N–B co-doped CDs. A study was conducted to determine the effects of temperature and reaction time throughout the microwave-assisted synthesis of CDs. These experiments were carried out using a standard pH of 5 and a concentration of 1 mg/mL of 3APBA.

Fluorescence spectra were obtained at constant time and varying temperatures of 120 °C, 140 °C, 160 °C, and 180 °C, and at constant temperature and varying times of 3, 5, 7, and 10 min for each case (Figure 3a). The results showed that the material’s optimal reaction time and temperature are 9 min and 140 °C. However, since the conditions of 3 min and 120 °C yielded similar results, it was decided to use these parameters to synthesize all batches used in this project. The decision to use these parameters was also made since the lower temperature and shorter reaction time would be more energy-efficient and cost-effective (Figure 3b). Overall, the optimum conditions for synthesizing N–B co-doped CDs are 3 min at 120 °C, pH 5, power 150 W, and a 1 mg/mL precursor concentration. These conditions should be used as a starting point for further optimization and experimentation to obtain the desired optical properties of CDs for specific applications.

Table 2 shows a comparison of the preparation of B and N co-doped CDs. Xiao and co-workers developed a solvothermal methodology using the conditions of 480 min at 160 °C in an autoclave, with 1 mg/mL of 3APBA as a precursor leading to a QY of 7% at 430 nm [44]. Following this, a household microwave oven was utilized as the first approach to obtain this type of co-doped nanomaterial. Tran and co-workers utilized boric acid and passion fruit juice in low concentrations to obtain CDs in 20 min at a temperature of 170 °C with 800 W of power, leading to a QY of 50% at 440 nm [46]. High concentrations of citric acid and boric acid in the presence of urea were used by Hsieh and co-workers [47] and Bian and co-workers [48] for time periods of 15 min and 4 min, respectively. For the first case, 180 °C with 720 W of power was used, leading to a QY of 30.2% at 450 nm; meanwhile, in the second case, researchers only controlled the approximately 900 W of power (given by a household microwave oven) and no QY was reported emitting at 460 nm. Finally, a variation in this procedure was published by Tian et. al, using ethylenediamine as a nitrogen source instead of urea. After 60 min of radiation at a temperature of 200 °C and 800 W of power, a QY of 66.5% was achieved at 446 nm [49]. In the case of this work, a similar QY was obtained at 375 nm, with the conditions of 3 min, 130 °C, and 150 W, using a monomodal microwave reactor because it enables full parameter control.

### 3.4. Fourier-Transform Infrared Spectroscopy and Raman Spectroscopy

**Figure 4:**
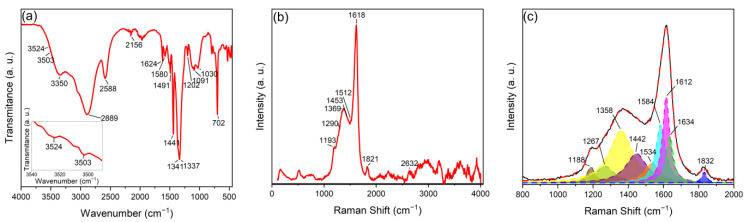
(**a**) FTIR spectra of the N–B co-doped CDs synthetized using the following conditions: 120 °C, 3 min, 150 W, and 1 mg/mL. Inset (**a**) shows FTIR spectra from 3450 cm^−1^ to 3550 cm^−1^ where the two vibrational modes of the primary amine are found. (**b**) The Raman spectra obtained with a 325 nm laser of N–B co-doped CDs synthetized using the following conditions: 120 °C, 3 min, 150 W, and 1 mg/mL, and (**c**) Raman spectra from 800 cm^−1^ to 2000 cm^−1^ with a deconvolution of nine signals of N–B co-doped CDs synthetized using the following conditions: 120 °C, 3 min, 150 W, and 1 mg/mL.

FTIR was used to investigate the functional groups in the internal structures and surfaces of the N–B co-doped CDs. The FTIR spectrum (Figure 4a) showed a N–H scissoring mode at 702 cm^−1^, indicating the presence of a primary amine [60]. This signal was supported by the peaks observed at 1337 cm^−1^, 1624 cm^−1^, 3503 cm^−1^, and 3524 cm^−1^, which corresponded to the stretching modes of C–N [60,61], bending modes of N–H [60], and two stretching modes of N–H for primary amines, respectively (see embedded graph in Figure 4a). Additionally, the signal at 2588 cm^−1^ was attributed to an overtone derived from the presence of stretching modes of primary amines [60], confirming the presence of this functional group in the nanostructure. A signal located at 2155 cm^−1^ corresponded to a stretching mode of C≡N. The peaks observed at 1030 cm^−1^, 1091 cm^−1^, 1201 cm^−1^, and 1341 cm^−1^ were associated with the vibrational deformation modes of B–O, stretching modes of C–B, bending modes of B–O–H, and stretching modes of B–O, respectively [32]. The peak observed at 3350 cm^−1^ corresponding to the stretching mode of O–H demonstrated the presence of the boronic acid group in the nanomaterial structure. The peaks observed at 1441 cm^−1^, 1491 cm^−1^, and 1580 cm^−1^ were characteristic of aromatic groups and corresponded to the bending modes of symmetric C=C, asymmetric C=C, and aromatic C=C, respectively. Therefore, the presence of an aromatic motif in the nanostructure can be confirmed. The findings from Raman and XPS spectroscopies, which are presented below, support this theory. In addition to corresponding to the stretching mode of C–N, the peak observed at 1337 cm^−1^ may also correspond to the stretching mode of B–N. This suggests that the material may be doped with a substitution in the graphene network in addition to having functional groups on the surface.

Raman spectroscopy was employed to investigate the structural composition and functionalization of B–N co-doped CDs functionalized with boronic acids and amines. The Raman spectra revealed significant multisignals, shedding light on the unique properties of the material. In the low-wavenumber region (below 1200 cm^−1^), various signals related to the co-doping of nitrogen and boron were observed. These signals predominantly correspond to breathing vibration modes. The D peak of nitrogen appeared at approximately 1193 cm^−1^, indicating the presence of nitrogen species within the N–B co-doped CDs [60]. Additionally, Raman shifts associated with pyridinic or pyrrolic nitrogen that modify the breathing vibration mode of the aromatic rings were observed around ~1000–1050 cm^−1^ [62]. The signals related to boron doping exhibited characteristics of sp^2^-hybridized boron at ~800–1000 cm^−1^ [62]. These multisignals collectively provide insights into the incorporation of nitrogen and boron species into the carbon matrix, with each peak corresponding to distinct vibrational modes within the breathing mode region [62]. Moving to the mid-wavenumber range (1200–1800 cm^−1^), the Raman spectra exhibited notable signals related to functional groups and carbon–carbon bonding. These signals primarily fall into the kekulé vibrational modes. The D band at 1369 cm^−1^ and the G band at 1618 cm^−1^ indicated the presence of disorder and sp^2^-hybridized carbon atoms within the N–B co-doped CDs. The intensity ratio of the D and G bands (I_D_/I_G_) was found to be approximately 0.439, indicating the presence of more sp^2^ than sp^3^ carbon atoms in the lattice. Furthermore, a signal associated with boronic acid stretching vibrations was observed at 1453 cm^−1^, while amine stretching vibrations appeared at 1512 cm^−1^. These grouped signals within the kekulé mode region suggest the successful functionalization of the N–B co-doped CDs with boronic acids and amines. In the high-wavenumber region (1800–4000 cm^−1^), a Raman signal at 1821 cm^−1^ indicated the presence of a C≡N bond within the N–B co-doped CDs. This signal falls into stretching vibration modes, which are characterized by stronger stretching of chemical bonds. The existence of the C≡N bond was validated by a stretching vibration peak at 1254 cm^−1^ in the FTIR spectra. Finally, multisignals corresponding to the region of the 2D band were found at 2632 cm^−1^. According to Maciel, the shape and the shift to the lowest frequencies of the signal is due to the combination of n-type and p-type doping, which is consistent with the presence of boron (electron-deficient) and nitrogen (electron-rich) [63].

The Raman spectra of the carbon dots were analyzed in depth using a deconvolution technique to identify and assign nine prominent peaks inside the 800–1800 cm^−1^ region: S_L_, S, D_S_, D, A_1_, A_2_, G_G_, D′, and C [62]. The S_L_ peak at 1188.14 cm^−1^ corresponds to the fundamental breathing mode of smaller polycyclic aromatic hydrocarbons (PAHs) and the secondary breathing mode of naphthalene. This suggests a multilayer PAH structure with defects and amorphous carbon on the surface. The S peak at 1267.84 cm^−1^ indicates the frustrated breathing mode of larger PAHs containing graphitic nitrogen and the secondary breathing mode of a seven-or-more-membered ring connected to graphitic nitrogen. This confirms the presence of aromatic rings and graphitic nitrogen within the multilayer PAH structure. The Ds peak at 1358.39 cm^−1^ is associated with disorder-induced Raman scattering resulting from structural defects, while the D peak at 1448.36 cm^−1^ represents the breathing mode of sp^2^ carbon atoms in disordered carbon materials. These findings suggest the presence of defects, amorphous carbon, or the multilayer PAH structure within the carbon dots. The A_1_ and A_2_ peaks at 1534.63 cm^−1^ and 1584.72 cm^−1^, respectively, are overtone and combination bands of the D peak, providing information about the degree of disorder, the extent of conjugation, and the presence of surface functional groups. The G_G_ peak at 1612.48 cm^−1^ is assigned to the G-band associated with graphene-like materials, indicating the presence of sp^2^ carbon atoms in the multilayer PAH structure. The D′ peak at 1634.64 cm^−1^ represents the breathing mode of sp^3^ carbon atoms in disordered carbon materials, potentially originating from heteroatomic dopants, amorphous carbon, or the multilayer PAH structure. The C peak at 1832.57 cm^−1^ indicates the presence of carbon–oxygen (C=O) bonds, which may result from the incorporation of carbonyl groups on the surface, consistent with the information obtained via XPS [64].

### 3.5. X-ray Photoelectron Spectroscopy

**Figure 5:**
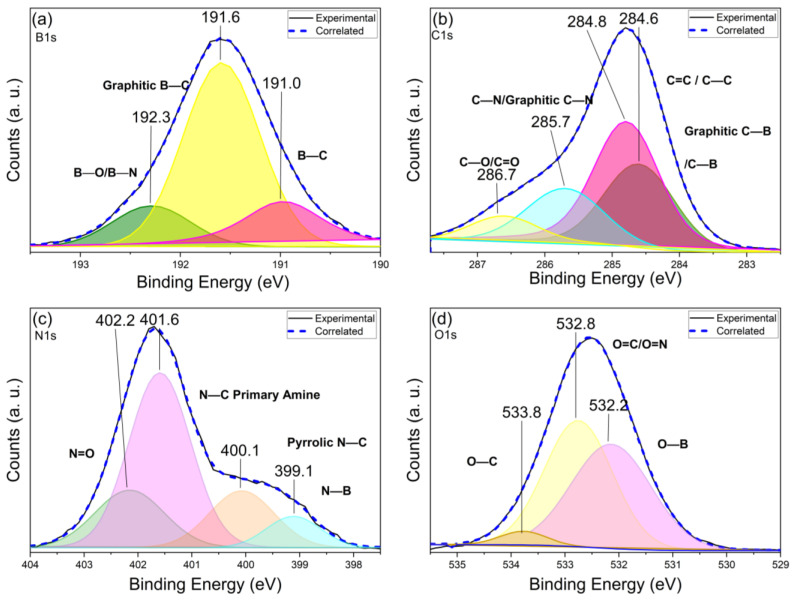
High-resolution XPS spectra of (**a**) B1s, (**b**) C1s, (**c**) N1s, and (**d**) O1s of N–B co-doped CDs synthetized with 120 °C, 3 min, 150 W, and 1 mg/mL.

Figure 5 shows the high-resolution XPS spectra for boron, carbon, nitrogen, and oxygen, respectively. The binding energies of 191 eV, 191.6 eV, and 192.3 eV were assigned to B–C [64], Graphitic C [65,66], and B–O/B–N bonds [67,68], respectively, indicating the presence of boronic acid groups on the surface of the nanomaterial and evidence of substitutional doping within the graphitic network (Figure 5a). The binding energies of 284.6 eV, 284.8 eV, 285.7 eV, and 286.7 eV (Figure 5b) were assigned to C–B/Graphitic B [68], C–C/C=C [65,69], C–N/C–Graphitic N [70,71], and C–O bonds [70], respectively. These results suggest the presence of an ordered graphitic-like carbon structure. Furthermore, evidence of the formation of the primary amine and boronic acid (C–B) bonds, as well as substitutional doping with nitrogen and boron, was observed. The binding energies of nitrogen were consistent with those obtained for carbon and boron, indicating substitutional doping in the graphitic network. The binding energies of 399.1 eV for N–B [67] and 400.1 eV for pyrrolic N–C [65] further support this conclusion, as does the binding energy of 401.6 eV assigned to primary amine N–C [65,71] (Figure 5c). The binding energy of 402.2 eV assigned to N=O [71] suggests the formation of oxides on the surface of the nanomaterial. Finally, the binding energies for oxygen (Figure 5d) confirm the presence of boronic acid groups with the signal of 532.2 eV for O–B [67], as well as evidence of oxide formation on the surface of the nanomaterial with the signal of 532.8 eV for O=C/O=N [72]. The binding energy of 533.8 eV was assigned to the hydroxyl groups surrounding the CDs [72].

### 3.6. Theoretical Calculations and Raman Spectra Correlation

**Figure 6:**
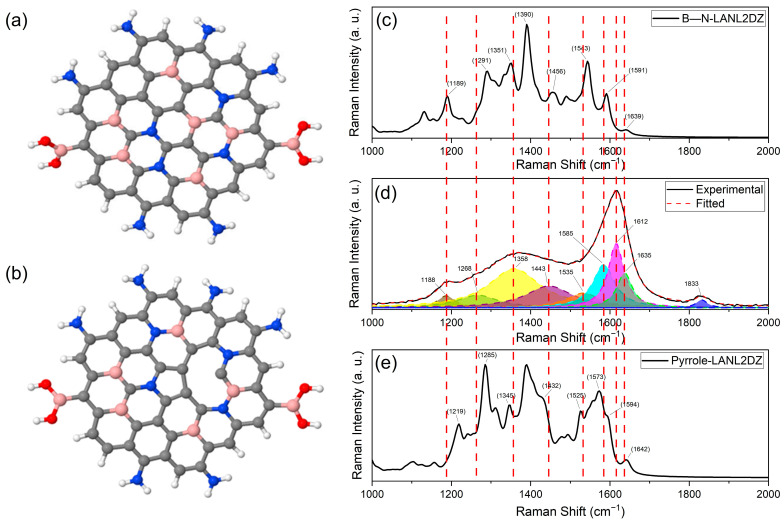
Theoretical structural arrays (**a**) B–N-LALN2DZ and (**b**) Pyrrole-LALN2DZ. Calculated Raman spectra of the theoretical structural arrays (**c**) B–N-LALN2DZ and (**e**) Pyrrole-LALN2DZ, and (**d**) Raman Spectra of the N–B co-doped CDs with nine deconvoluted signals.

Figure 6a,b display the low-energy configurations of two proposed structural arrays of B–N co-doped CDs, B–N-LALN2DZ and Pyrrole-LALN2DZ, respectively, based on empirical data. B–N-LALN2DZ (Figure 6a) was formulated considering the presence of amino and boronic acids near the particle, with boron and nitrogen atoms substituting carbon atoms, along with a B–N bond. Pyrrole-LALN2DZ (Figure 6b) extends the previous array by incorporating a vacancy and a pyrrole group.

The theoretical Raman spectra of B–N-LALN2DZ (Figure 6c) exhibit seven representative signals corresponding to those detected in the experimental data. The frequencies at 1833 cm^−1^ (C assignment, carbonyl stretching mode) and 1612 cm^−1^ (G_G_ assignment, stretching modes of PAHs) from the experimental data are not found in the theoretical spectrum due to the lack of carbonyl groups and symmetry loss from boron and nitrogen doping.

The theoretical Raman spectra of Pyrrole-LALN2DZ (Figure 6e) illustrate eight peaks that align with the experimental signals. However, the C assignment associated with the carbonyl stretching mode is absent in the spectrum due to the exclusion of C=O bonds in the theoretical structural array.

Table 3 compiles the data from theoretical and experimental Raman spectra, comparing them with a nine-peak model of doped PAHs proposed by Sabin et al. [62]. It includes the range of assignments, peak names, and Raman shifts for each signal in the experimental spectrum. Both theoretical models present shifts due to the influence of heteroatomic doping on the electronic nature of the array. For example, the boron atom’s less electronegative nature can increase electron density in adjacent bonds, strengthening them and causing a blue shift. In contrast, nitrogen’s higher electronegativity can redistribute electron density, weakening nearby bonds and resulting in a redshift.

These models, as first approximations, offer valuable insights into the structure of B–N co-doped CDs. However, their finite nature might fail to account for some vibrational modes observed in the experimental Raman spectra, like interlayer interactions. Moreover, edge effects in these models, where atoms on the particle’s periphery could behave differently compared with those in a larger and more realistic model, might impact the electronic structure and vibrational modes, affecting the calculated Raman spectra. Nonetheless, these models provide a reasonably good fit with the experimental data. Pyrrole-LALN2DZ appears to provide a more accurate representation of the system due to its structural array, impurities, vacancies, specific bonds, functional groups, and the alignment of peaks with experimental data.

## 4. Conclusions

In this work, we developed nitrogen and boron co-doped and functionalized carbon dots using a monomodal microwave-assisted synthesis approach. The uniform size distribution of these B–N co-doped CDs has a mean diameter of 10.90 ± 3.39 nm and was confirmed using TEM analysis. The analysis of HRTEM micrographs revealed a 0.24 nm interplanar distance, which is higher than the (100) crystalline distance of 0.21 nm of graphite. This discrepancy is attributed to the presence of heteroatomic doping. The distinct optical properties of B–N co-doped CDs were revealed using UV–vis absorption and fluorescence spectroscopy, where π–π* and n–π* transitions were identified, and an emission peak at 375 nm with excitation at 295 nm was observed with a QY of 56.7%. Precise control of synthesis parameters led to these optimal properties, with a reaction time of 3 min, a temperature of 120 °C, a microwave power of 150 W, a pH of 5, and a precursor concentration of 1 mg/mL, resulting in the highest fluorescence emission intensity. Advanced spectroscopic methods further verified the successful doping and functionalization of these quantum dots. The FTIR, Raman, and XPS spectra methods confirmed the presence of boronic acid and amine functional groups, an ordered graphitic-like carbon structure, and the successful incorporation of nitrogen and boron as dopants. Theoretical Raman spectra calculations were compared with the deconvoluted experimental data and it was found that both models present similarities with the experimental Raman spectra. The Pyrrole-LALN2DZ model seems to fit more with the complexity of the experimental results. Nevertheless, further investigations will be conducted to elucidate the in-depth vibrational behavior of the B–N co-doped CDs. This significant accomplishment provides a promising basis for the potential utilization of these N-B co-doped CDs in different applications, such as fluorescent probes for cellular lines. Moreover, N-B co-doped CDs have the capacity to be applied in fluorescent recognition methods for a broad range of analytes due to the capacity of this material to 1: change its surface area with pH changes and 2: be covalently functionalized due to the presence of amine and boronic acid groups surrounding the particles. This allows us to add other molecules to make specific recognitions via covalent bonds or weak interactions. Also, boronic acid groups are well known to interact covalently with 1,3-diols in water pollutants.

## Figures and Tables

**Table 1:** Parameters of the microwave-assisted synthesis of the B–N co-doped CDs.

Time	T (°C)	Time	T (°C)	Time	T (°C)	Time	T (°C)	pH	C (mg/mL)	Power
3	120	3	140	3	160	3	180	5	1	150 W
5	120	5	140	5	160	5	180	5	3	150 W
7	120	7	140	7	160	7	180	5	5	150 W
9	120	9	140	9	160	9	180	5	10	150 W

**Table 2:** Comparison of N and B co-doped CDs using hydrothermal and microwave (MW) techniques.

Precursors	Concentration	Type of Reactor	Time (min)	Temperature (°C)	Power (W)	Ex/Em (nm)	QY%	References
3-aminophenylboronic acid	1 mg/mL	Solvothermal	480	160	N/A	350/430	7	[44]
boric acid/Passion fruit juice	150 mg/10 mL	MW-Multimodal	20	170	800	360/440	50	[46]
Citric acid/urea/boric acid	10 g, 10 g, 5 g	MW-Multimodal	15	180	720	360/450	30.2	[47]
Citric acid/urea/boric acid	1.093 g, 1 g, 1g	MW-Multimodal	4	N/A	~900	360/460	N/A	[48]
Citric acid/ethylenediamine/boric acid	N/A	MW-Multimodal	60	200	800	350/446	66.5	[49]
3-aminophenylboronic acid	1 mg/mL	MW-Monomodal	3	130	150	295/375	56.7	This Work

**Table 3:** Peak assignment of the B–N co-doped CDs and the theoretical structural arrays, B–N-LALN2DZ and Pyrrole-LALN2DZ, and the shifts of each of the signals.

Reference Assignment (cm^−1^) [62]	Peak Name	Experimental Assignment (cm^−1^)	Theoretical Assignment (cm^−1^) (B–N-LALN2DZ)	Theoretical Assignment (cm^−1^) (Pyrrole-LALN2DZ)	Shift B–N-LALN2DZ (cm^−1^)	Shift Pyrrole-LALN2DZ (cm^−1^)
900–1075	S_L_	1188	1189	1219	+1	+31
1150–1200	S	1268	1291	1285	+23	+17
1250–1300	D_S_	1358	1351	1345	−7	−13
1340–1380	D	1443	1456	1432	+13	−11
1400–1460	A_1_	1535	1543	1525	−8	−10
1480–1550	A_2_	1585	1591	1573	+6	−12
1570–1600	G_G_	1612	-	1594	-	−18
1605–1650	D’	1635	1639	1642	+4	+7
1750–1800	C	1833	-	-	-	-

## Data Availability

Not applicable.
